# Ramsay Hunt syndrome: long-term facial palsy outcome assessed face-to-face by three different grading scales and compared to patient self-assessment

**DOI:** 10.1007/s00405-020-06251-w

**Published:** 2020-08-03

**Authors:** Mervi Kanerva, Sanna Jones, Anne Pitkaranta

**Affiliations:** 1 Department of Otorhinolaryngology, Head and Neck Surgery, Helsinki University Hospital and University of Helsinki, PO Box 263, 00029 Helsinki, Finland; 2 Occupational Health, Helsinki, Finland

**Keywords:** House–brackmann grading system, Facial nerve grading system 2.0, Sunnybrook facial grading system, Facial paralysis, Herpes zoster oticus, Facial nerve

## Abstract

**Purpose:**

To determine the long-term facial palsy outcome of Ramsay Hunt Syndrome by face-to-face grading by House–Brackmann Grading System, Facial Nerve Grading System 2.0, and Sunnybrook Facial Grading System concomitantly. To compare the applicability of the grading scales. To compare patients’ self-assessed facial palsy outcome results to gradings performed by the investigator. To compare the face-to-face assessed facial palsy outcome to the initial palsy grade.

**Methods:**

Fifty-seven patients self-assessed their facial palsy outcome and came to a one-time follow-up visit. The palsy outcome was graded by one investigator using the three above-mentioned grading systems concomitantly. The median time from syndrome onset to follow-up visit was 6.6 years.

**Result:**

A good long-term face-to-face assessed palsy outcome was enjoyed by 84% of the patients. Trying to assess only one House–Brackmann grade to represent the palsy outcome was impossible for most patients. Facial Nerve Grading System 2.0 worked better, but needed adjustments and certain sequelae findings needed to be neglected for it to be executable. The Sunnybrook system worked the best. Nearly 20% of the patients assessed themselves differently from the investigator: both better and worse.

**Conclusion:**

The Sunnybrook scale was the most applicable system used. With antiviral medication, the outcome of facial palsy in Ramsay Hunt syndrome starts to resemble that of Bell’s palsy and emphasizes the importance of recognizing the syndrome and treating it accordingly. The results give hope to patients instead of the gloomy prospects that have stigmatized the syndrome.

## Introduction

Ramsay Hunt syndrome (RHS) involves peripheral facial palsy (FP) and herpes blisters in the head and neck area. In addition to the facial nerve (cranial nerve VII), the vestibulocochlear nerve (cranial nerve VIII) is frequently affected, which causes hearing loss, tinnitus, and/or vertigo [1]. All cranial nerves (V, VIII, IX, X, more rarely III, XI, XII) and cervical nerves (C2, C3, C4) that communicate with facial nerve can be affected [2–4]. The cause of RHS is reactivation of latent varicella-zoster virus (VZV) [1, 5, 6]. The incidence of RHS has varied among studies and has been estimated to be from 1 to 5/100,000/year or 4% to 18% of all FP cases [1, 7–10].

Even in one-third of RHS cases, blisters follow FP [10, 11] making the diagnosis of RHS impossible at the time of FP onset. Clinicians must be alert to remember the syndrome and inform patients of this possibility.

Published studies on RHS FP outcomes are scarce, including mostly retrospective chart studies, and lacking long follow-up periods; few follow the patients even up to 1 year [7, 12, 13]. The only reported prospective study including RHS patients with 1-year follow-up is from prior to when medical treatment was offered to these patients [7]. With retrospective studies and experience in relation to medications used in shingles, it is a widely accepted view that antiviral medication combined with corticosteroids is beneficial in RHS [14–17].

At present, we lack a reliable way to grade FP. Objective methods are being developed, but are not yet widely clinically used. The most used subjective method is the House–Brackmann grading scale (HB) [18], with six grades, where I is normal facial function and VI total paralysis. Each grade represents a certain voluntary movement status (as one for the whole face, not graded regionally), resting symmetry, and synkineses as defined in the scale instructions. HB is a gross scale, not originally intended for specifying the movements of different facial areas or delicate sequelae. Therefore, it is known to be poorly suitable to grade precise and detailed information of FP or record FP surgery outcomes [19–21]. A revised version, Facial Nerve Grading System 2.0 (FNGS) [22], was developed, allowing voluntary movements of the different facial areas and synkineses to be assessed separately. Symmetry at rest was still linked to voluntary movement assessment. FNGS coverage amongst published studies has been scant.

The Sunnybrook facial grading system (SB) [23, 24], on the other hand, seems to have grown in popularity amongst investigators [20, 25]. It is a regional weighted system, that takes into consideration the resting symmetry of the face, the degree of voluntary movement, and also synkineses regionally. The composite score varies from 0 to 100, where 0 stands for total paralysis and 100 for normal facial function.

The first aim of this study was to determine the long-term FP outcome of RHS by face-to-face grading by using the three above-mentioned grading scales concomitantly. The second aim was to compare the applicability of the grading scales and the results received. Third, we wanted to know whether patient self-assessed FP outcome results varied from gradings performed by the investigator. Finally, our aim was to compare the face-to-face assessed FP outcome grade to the initial FP grade in association with the medication patients received and medication start time at RHS onset.

## Materials and methods

A computer search from January 1996 to January 2013 was performed in our tertiary referral center: ICD codes for shingles with any complication or supplementary diagnosis with concomitant FP diagnosis were used. Patients with varicella-zoster blisters in the head and neck area within 1 month prior or after, or concomitantly with, palsy onset were included in the study.

Patients were sent a questionnaire about their RHS [11]. They were asked to self-assess their RHS FP outcome and were invited to come to one follow-up visit, where their facial function was graded by the corresponding author with HB, FNGS, and SB concomitantly.

We gathered information from the patient records on initial FP grade, what medication was used and when it was started in relation to RHS onset.

The study group was composed of patients fulfilling the diagnosis requirements, with a minimum of one year from the RHS onset, answering the questionnaire, and being able to come to a follow-up visit.

FP grading methods at initial visit varied in the patient records, and both HB and SB grading scales had been used. For study purposes, to be able to compare SB results to HB and FNGS results, SB grades were converted to HB grades according to a conversion table: SB 100 converting to HB I; SB 70–99 to HB II; SB 43–69 to HB III; SB 26–42 to HB IV; SB 13–25 to HB V; and SB 0–12 to HB VI [26].

The Helsinki University Hospital Ethics Committee approved the study and institutional research approval was granted. All patients gave their written informed consent.

We used SPSS (IBM Corp., Armonk, NY, USA) versions 22–24 for statistical analyses. We used descriptive statistics to summarize the frequencies, proportions, means or medians, and ranges.

## Results

### Patients

Computer search revealed 120 RHS patients: 71 women, 49 men. At the time of the study, 8 of the 120 patients had died and four could not be reached. The remaining 108 patients were sent a questionnaire about their RHS and were invited for one follow-up visit for facial function grading. Of these, 81 answered the questionnaire (52 women, 29 men) and 57 (41 women, 16 men) came for a follow-up visit. A caregiver of additional three patients informed that due to the patient’s illnesses, the patient was no longer capable of answering the questionnaire or to come for a follow-up visit.

We have previously published the results of the retrospective chart study (120 patients) and the patient questionnaire study (81 patients): medical treatment, blister location and time to FP, hearing loss and its outcome, other adjoining symptoms, and outcome of FP on grounds of patient charts, and patient self-assessment [11]. In the current study, we concentrated on the face-to-face assessed long-term FP outcome of those 57 patients coming to the follow-up visit. Other characteristics of their RHS were included in the results of the previous study and are not repeated here.

Altogether 26/57 (46%) patients experienced FP on the right, and 31/57 (54%) on the left. Age median at RHS onset was 48.8 years (range 6.9–79.7): for women 50.2 (range 19.9–79.7) for men 45.5 (range 6.9–71.5). Two of the patients were 16 years or younger at RHS onset. The median follow-up time was 6.6 years (range 1–17.1). 6 of the 57 patients (10.5%) had an illness or medication that made them immunocompromised and could have predisposed the patient to RHS. No one had a history of previous RHS or relatives with RHS. One patient had experienced FP and total recovery a long time before. Six patients had a relative with FP history.

### The applicability of the grading scales

The use of FNGS and HB revealed inconsistencies. With HB, each grades I–VI includes definitions for what the grade stands for with respect to voluntary movement in the forehead, eye, and mouth as well as the level of symmetry at rest, and synkineses; all of these combined to one representing grade. Nevertheless, voluntary movement recovery levels in different parts of the face differ sometimes greatly. In this study, placing the patient to only one HB grade was impossible in many cases. Even if one compromised voluntary movement grade was set between forehead, eye, and mouth, the resting symmetry and/or synkineses status was still many times in disagreement with the grade best representing the voluntary movement status. Even though HB grading was carried out throughout the study with additional notes to mark the discrepancies, the results are not presented here, since 37/57 (65%) of the patients would have needed two or more HB grades to represent their FP outcome.

FNGS worked better than HB, but the symmetry at rest results were in disagreement with the voluntary movement results (to which they are anchored in this grading system) with 17/57 (30%) of the patients. The voluntary movement outcome was considered more important and asymmetry at rest had to be disregarded in these cases, resulting in better outcome results than in reality.

SB was the most applicable of the three grading scales used since it allows regional assessment of all variables; voluntary movement, symmetry at rest, and synkineses.

### Medical treatment

At RHS onset, all but one of the 57 patients were prescribed antiviral medication; 28 (49%) were also prescribed concomitant corticosteroids (Table 1). One patient admitted not taking either of the prescribed medicines and was placed in the “no medication” group for the outcome results.

**Table 1:** Long-term outcome of Ramsay Hunt syndrome facial palsy compared to medication and its star time: initial facial palsy grade (known for 28 patients), follow-up visit facial palsy grade (57 patients), and patient’s self-assessment (57 patients)

Medication at Ramsay Hunt syndrome onset	Number of patients	Medication onset time from Ramsay Hunt diagnosis/patient number; initial HB grade → follow-up visit assessed HB grade^†^/patient number; patient self-graded outcome^‡^/patient number			
		≤ 24 h	≤ 48 h	≤ 72 h	> 72 h
Valaciclovir 1 g × 3 × 7 orally	9	8; HB VI → HB I/1; 2/1 HB ? → HB I/3; 1/2, 2/1 HB ? → HB II/3; 2/3 HB III → HB II/1; 1/1		1; HB ? → HB I/1; 1/1	
Valaciclovir orally, dosing unclear	1	1; HB V → HB II/1; 2/1			
Valaciclovir 1 g × 3 × 7 orally + corticosteroid^¶^	24	22; HB II → HB II/1; 1/1 HB III → HB I/2; 1/2 HB III → HB II/1; 4/1 HB IV → HB II(I)/1; 1/1 HB IV → HB II/2; 2/2 HB V → HB II/1; 2/1 HB V → HB III/1; 3/1 HB VI → HB II/1; 3/1 HB VI → HB IV/1; 3/1 HB ? → HB I/4; 1/4 HB ? → HB II(I)/1; 1/1 HB ? → HB II/5; 2/4, 3/1 HB ? → HB IV(V)/1; 3/1	1; HB II → HB I/1; 1/1		1; HB V → HB III/1; 3/1
Acyclovir 800 mg × 5 × 7 orally	4	3; HB III → HB I/1; 2/1 HB IV → HB III/1; 2/1 HB ? → HB I/1; 1 /1			1; HB VI → HB II/1; 2/1
Acyclovir orally, dosing under 800 mg × 5 × 7 or unclear	4	3; HB IV → HB I/1; 1/1 HB ? → HB II/1; 1/1 HB ? → HB II(I)/1; 2/1	1; HB ? → HB II/1; 3/1		
Acyclovir under 10 mg/kg/day × 3 or unclear intra venous	1	1; HB II → HB I/1; 1/1			
Acyclovir dosing under 800 mg × 5 × 7 orally or under 10 mg/kg/day × 3 intra venous + corticosteroid^¶^	2	2; HB V → HB III/1; 2/1 HB II → HB I/1; 1/1			
Acyclovir 10 mg/kg/day × 3 intra venous	8	7; HB V → HB II/2: 2/1, 3/1 HB VI → HB III/1; 2/1 HB ? → HB II/4; 1/1, 2/2, 4/1			1; HB II → HB II/1; 3/1
Acyclovir 10 mg/kg/day × 3 intra venous + corticosteroid^¶^	2	1; HB II → HB II(I)/1; 2/1			1; HB ? → HB IV/1; 3/1
No medication	2				2; HB ? → HB II/1; 2/1 HB ? → HB III(IV)/1; 3/1

^†^Initial facial palsy grade was available from 28/57 patient records, graded by House-Brackmann scale (HB) or Sunnybrook scale (SB), latter converted here to HB grades. Outcome was graded by SB and Facial Nerve Grading System 2.0 (FNGS) converted here to HB grades. When SB and FNGS differ, the latter is in parentheses

^‡^Facial palsy outcome by patient’s own assessment: 1 = totally recovered; 2 = slight sequelae; 3 = obvious sequelae; 4 = severe sequelae

^¶^Corticosteroid usually Prednisolon 60 mg/day with tapering dosing 10 mg/day after 5 days for 10 days or Medrol 32 mg or 64 mg/day for 7–10 days

Of the 57 patients, 50 (88%) received their medication within 48 h, 1 (1.8%) within 72 h, and 6 (10.5%) over 72 h from the onset of RHS or no medication at all (Table 1).

### Comparison of long-term FP outcome to initial medication

The patient number in this study was too small to draw statistically reliable conclusions whether the type or dosing of the used medication played any role in the outcome of FP. In our previous study [11], the outcome stayed the same as long as the medication was started within 72 h of the RHS onset. The outcome was worse with those receiving no medication or starting the medication over 72 h from RHS onset.

The same result trend could be seen in this study (a subgroup of the previous study; individual patient outcomes not shown). The number of patients receiving their medication later than 72 h or not taking any medication at all was low, 6/57 (10.5%), but none recovered totally and 3/6 (50%) recovered to having only slight sequelae.

### Comparison of the long-term FP outcome to initial FP grade

Of these 57 graded patients, 28 (49%) had a mention of the FP grade at the initial visit in their charts. There were 12 patients with HB V–VI; 5 HB IV; 5 HB III; and 6 HB II (Table 1).

The face-to-face assessment SB outcome results have been converted here to HB grades (see Methods) to be able to compare to the base line HB grades. From HB V–VI, 7/12 (58.5%) patients recovered to HB I–II, 4 even with an SB grade of 96 (of 100) or better, which is an excellent result; 4/12 (33.3%) to HB III; and 1/12 (8.3%) to HB IV. From HB IV, 4/5 (80%) recovered to HB I–II; 1/5 (20%) to HB III. From HB III and HB II, all 11 patients recovered to HB I–II. Those initially with mild palsy of HB II recovered all to level 90 or better with SB grading (Table 1).

### Long-term outcome of all 57 patients

SB face-to-face long-term FP outcome voluntary movement recovery results are seen in Table 2 and the final total scores in Table 3. Converting the SB total score results to HB grade: 17/57 (30%) recovered to HB grade I; 31/57 (54%) to HB II; 6/57 (11%) to HB III; and 3/37 (5%) to HB IV. Of those 33 patients that had total voluntary movement recovery (SB 100), 12/33 (36%) had slight synkinesis that affected their total score in SB to be worse than 100 (Table 3). Four of these patients also had slight asymmetry of the face at rest. An additional 4/33 (12%) patients with total voluntary movement recovery had only asymmetry at rest, and no synkinesis.

**Table 2:** Sunnybrook voluntary movement scores for 57 follow-up patients

Sunnybrook score	Patient number/%
100	33/57.9
90–99	10/17.5
80–89	6/10.5
70–79	2/3.5
60–69	4/7.0
50–59	1/1.8
40–49	1/1.8

**Table 3:** Sunnybrook total scores for 57 follow-up patients

Sunnybrook score	Patient number/%
100	17/29.8
90–99	20/35.1
80–89	6/10.5
70–79	5/8.8
60–69	2/3.5
50–59	3/5.3
40–49	2/3.5
30–39	1/1.8
20–29	1/1.8

Altogether, synkineses were detected by SB in 34/57 (60%) patients, asymmetry at rest in 23/57 (40%), and both or either one in 38/57 (67%).

Overall, 84% of the patients with SB experienced a good outcome (SB 70–100, converting to HB I–II).

FNGS (used as described previously: e.g. disregarding asymmetry when in disagreement with the voluntary movement outcome) resulted in 21/57 (37%) total recoveries (grade 1); 27/57 (47%) good recovery (grade 2); 5/57 (9%) grade 3; 3/57 (5%) grade 4; and 1/57 (2%) grade 5. Altogether, a good recovery (grades 1 and 2) was achieved for 48/57 (84%) patients.

Thus, SB and FNGS (with adjustments) resulted in 30% and 37%, respectively, total recovery and both in 84% good recovery outcome results.

### Comparison of patient self-assessment to grading by the investigator

Of those 17 patients, who were assessed by the investigator to be totally recovered, three (18%) assessed themselves as incompletely recovered, reporting slight sequelae; tightness of the face, occasional numbness of the face, asymmetry, and functional defects (Table 1). In addition, two patients who were placed in HB grade II (SB 70–99; their SB grades 72 and 89) assessed themselves having severe sequelae (Table 1). Altogether, 5/57 (9%) self-assessed themselves worse recovered than the investigator’s assessment. On the other hand, 6/57 (10%), who were graded to have slight synkinesis, slight asymmetry at rest, and/or slight motor dysfunction, had considered themselves as totally recovered (Table 1). Thus, 11/57 (19%) of the self-assessment FP outcome results differed from the investigator’s assessment.

## Discussion

### Grading

All studies assessing FP outcome have the same weakness and difficulty of lacking a reliable method of grading [20]. All grading systems in wider clinical use today are subjective. Objective methods are not yet in everyday use or are only usable for a certain facial region [27–29]. Many of the studies on grading methods have understandably also used video recordings of the patients. Researchers have shown, however, that grading the facial function face-to-face with the patient as compared to grading from a video affects the results obtained, especially concerning the grading of synkinesis [30]. Thus, there is a definite need for an affordable, easy to use, reliable, and objective grading method.

We used three grading scales concomitantly to compare their clinical suitability. Trying to assess only one HB grade to represent the FP outcome was impossible with most patients. This observation leads to critically view the previously reported HB outcomes of FP studies. There is no way of knowing how systematically synkineses and resting symmetry have been included in these assessments. By saying this, we want to draw the reader’s attention to the fact, that using FNGS, and especially SB grading, we are most probably being more precise and getting worse results than if we used HB in a “liberal way”, assessing the patient with a compromised HB grade and ignoring conflicting synkinesis or asymmetry findings. The use of HB mostly reflects patient’s voluntary movement recovery and is a compromise between the different areas of the face. We also claim, that the HB grading system has been mainly used as a linear grading from I to VI, with assessors not so much paying attention to what each grade’s definition describes and demands. This thought has arisen from the observation that if those definitions are followed, the use of HB grading becomes impossible because of the discrepancy of findings. HB not being specific enough and recognizing that originally it was not even meant to do so, have been previously shown by our own studies [19, 26] and by many colleagues as well [20, 21, 31].

Although FNGS worked better in this study than HB, the use of FNGS needed adjustments and inevitable disregard of certain findings. Previously, it has been observed that there are no published studies on intraobserver reliability of FNGS [20].

Based on the results acquired from this and the previous studies [20, 25], we suggest that of these three grading scales, SB should be the one recommended to be used.

In their self-assessment, part of the patients assessed their FP outcome differently from the assessment by the investigator. The face can look and function symmetrically, but still feel stiff or weak. This phenomenon of discrepancy between self-assessments vs investigator gradings has been shown in the previous studies [32, 33]. Hence, every patient’s illness and needs are unique, no matter how similar they look from the outside.

### FP outcome

In our study, the long-term FP outcome “good recovery” was obtained by 84% of the patients. The result was very good, but still in concordance with studies where medical therapy (antivirals and corticosteroids) has been used [4, 12–14, 34–36]. Even though in our study almost all patients received antiviral therapy, only half received simultaneous corticosteroid treatment. The addition did not seem to affect the FP outcome. The patient number was too small to draw further conclusions about the different medical treatment modalities. These outcome results far exceed those seen prior to the application of antiviral medication [7, 8]. With these outcome results, the previously considered, much worse outcome of RHS FP compared to Bell’s palsy does not seem to be so drastic anymore. If we study Bell’s palsy patients (that have received corticosteroid treatment) meticulously after a minimum of one year from the palsy onset with the SB grading method, about 30% will have sequelae [37]. We could say, that the outcome results of Bell’s palsy and RHS FP have converged with each other and the diagnosis of RHS is no longer so devastating to the patient as before.

## Strengths and limitations

The strength of this study was the novel follow-up visit assessment by one investigator of the long-term (median follow-up time over 6 years) outcome of RHS FP. We also had a fairly large group of RHS patents answer the questionnaire and come to the visit. A strength was also that the FP grade evaluations at the visit were done by only one investigator, so the gradings were comparable to each other.

A limitation was that FP gradings at RHS onset were not standardized and were collected from patient records retrospectively. The time the patient was first seen and evaluated after FP onset also varied amongst patients. The low patient number unfortunately allowed only suggestive conclusions to be drawn about the initial medical treatment and FP outcome.

## Conclusions

Is it time to wean ourselves from the use of the HB grading system; do we get a false sense of accuracy, even though the given grade is a result of compromise and individual interpretation? In this study, FNGS also needed ignorance of some of the sequelae to be executable. Of the three scales used, SB was the most applicable.

The results also emphasized the importance of recognizing RHS, since with antivirus medication the outcome of RHS FP starts to resemble that of Bell’s palsy and gives hope to patients afflicted by RHS.
